# Introducing *Infectious Disease Reports*: Past, Present and Future

**DOI:** 10.3390/idr12030024

**Published:** 2020-12-11

**Authors:** Nicola Petrosillo

**Affiliations:** Clinical and Research Department for Infectious Diseases, National Institute for Infectious Diseases “L. Spallanzani”, IRCCS-Via Portuense, 292-00149 Rome, Italy; nicola.petrosillo@inmi.it

The first two decades of the New Millenium have faced several and threatening problems in healthcare. Emerging viral outbreaks, including SARS in 2004, MERS-CoV in 2012, Ebola virüs disease in 2013-16 in West Africa and more recently in the Democratic Republic of Congo, and the recent SARS-CoV-2 pandemic, represent the most worrying menaces of the microbial world against Humankind.

While emerging viral pandemic infections threaten nations due to their fast spread, infectious diseases go beyond COVID-19. During the past two decades, we have been experiencing the rise of resistance to antimicrobials in microrganisms. Currently, the major drivers behind the occurrence and spread of antimicrobial resistance are represented by the use of antibiotics in human and veterinary settings, and in the environment, mainly for agricultural purposes. The healthcare setting represents the melting pot and the sounding board for several multidrug-resistant organisms; indeed, while antimicrobial overuse/misuse determines ecological pressure on bacteria and contributes to the emergence and selection of AMR, poor infection prevention and control procedures, overcrowding, understaffing, and limited or lacking antimicrobial stewardship programmes greatly contribute to the growth and the spread of life threatening organisms.

Before all public and healthcare attention was focused on COVID-19, tackling antimicrobial resistance was the main issue in infectious diseases and healthcare. Initiatives have been undertaken on different fronts, including a One Health approach, public awareness, education, rapid diagnostics, sanitation and hygiene, epidemiology and surveillance, resources, and novel drugs. When “the dust” created by COVID-19 settles a little, we will face the same infective problems that we left behind. 

*Infectious Disease Reports* (*IDR*), launched in 2009 by PagePress publications, is currently managed by MDPI, an organization with great experience in Open Access science publishing. As the current Editor-in-Chief, my vision (and my expectation) for *IDR* is to encourage the Infectious Disease community and all the other specialties dealing with infections to share information, evidence, experiences and knowledge in the broadest way possible, in order to grasp and address rapidly evolving global changes and challenges in the field of infections, microbiology and host immunology, prevention and control, diagnostics, treatment and, moreover, global health.

The Editorial Board of *IDR* has both young, emerging and well-renowned infectious disease experts ensuring fast, high-level peer review. Fast online publication, proposals for Special Issues, and other promoting initiatives will help *IDR* increase in visibility and quality. I look forward to positioning *IDR* among the major infectious disease journals, providing solid help in the fight against infectious diseases worldwide.

